# Functional Organization of the Sympathetic Pathways Controlling the Pupil: Light-Inhibited and Light-Stimulated Pathways

**DOI:** 10.3389/fneur.2018.01069

**Published:** 2018-12-18

**Authors:** Elemer Szabadi

**Affiliations:** Developmental Psychiatry, Queen's Medical Centre, University of Nottingham, Nottingham, United Kingdom

**Keywords:** pupil, sympathetic, light, locus coeruleus, species difference, dorsal raphe nucleus, Edinger-Westphal nucleus, arousal

## Abstract

Pupil dilation is mediated by a sympathetic output acting in opposition to parasympathetically mediated pupil constriction. While light stimulates the parasympathetic output, giving rise to the light reflex, it can both inhibit and stimulate the sympathetic output. Light-inhibited sympathetic pathways originate in retina-receptive neurones of the pretectum and the suprachiasmatic nucleus (SCN): by attenuating sympathetic activity, they allow unimpeded operation of the light reflex. Light stimulates the noradrenergic and serotonergic pathways. The hub of the noradrenergic pathway is the locus coeruleus (LC) containing both excitatory sympathetic premotor neurones (SympPN) projecting to preganglionic neurones in the spinal cord, and inhibitory parasympathetic premotor neurones (ParaPN) projecting to preganglionic neurones in the Edinger-Westphal nucleus (EWN). SympPN receive inputs from the SCN via the dorsomedial hypothalamus, orexinergic neurones of the latero-posterior hypothalamus, wake- and sleep-promoting neurones of the hypothalamus and brain stem, nociceptive collaterals of the spinothalamic tract, whereas ParaPN receive inputs from the amygdala, sleep/arousal network, nociceptive spinothalamic collaterals. The activity of LC neurones is regulated by inhibitory α_2_-adrenoceptors. There is a species difference in the function of the preautonomic LC. In diurnal animals, the α_2_-adrenoceptor agonist clonidine stimulates mainly autoreceptors on SymPN, causing miosis, whereas in nocturnal animals it stimulates postsynaptic α_2_-arenoceptors in the EWN, causing mydriasis. Noxious stimulation activates SympPN in diurnal animals and ParaPN in nocturnal animals, leading to pupil dilation via sympathoexcitation and parasympathetic inhibition, respectively. These differences may be attributed to increased activity of excitatory LC neurones due to stimulation by light in diurnal animals. This may also underlie the wake-promoting effect of light in diurnal animals, in contrast to its sleep-promoting effect in nocturnal species. The hub of the serotonergic pathway is the dorsal raphe nucleus that is light-sensitive, both directly and indirectly (via an orexinergic input). The light-stimulated pathways mediate a latent mydriatic effect of light on the pupil that can be unmasked by drugs that either inhibit or stimulate SympPN in these pathways. The noradrenergic pathway has widespread connections to neural networks controlling a variety of functions, such as sleep/arousal, pain, and fear/anxiety. Many physiological and psychological variables modulate pupil function via this pathway.

## Introduction

**Figure 1:**
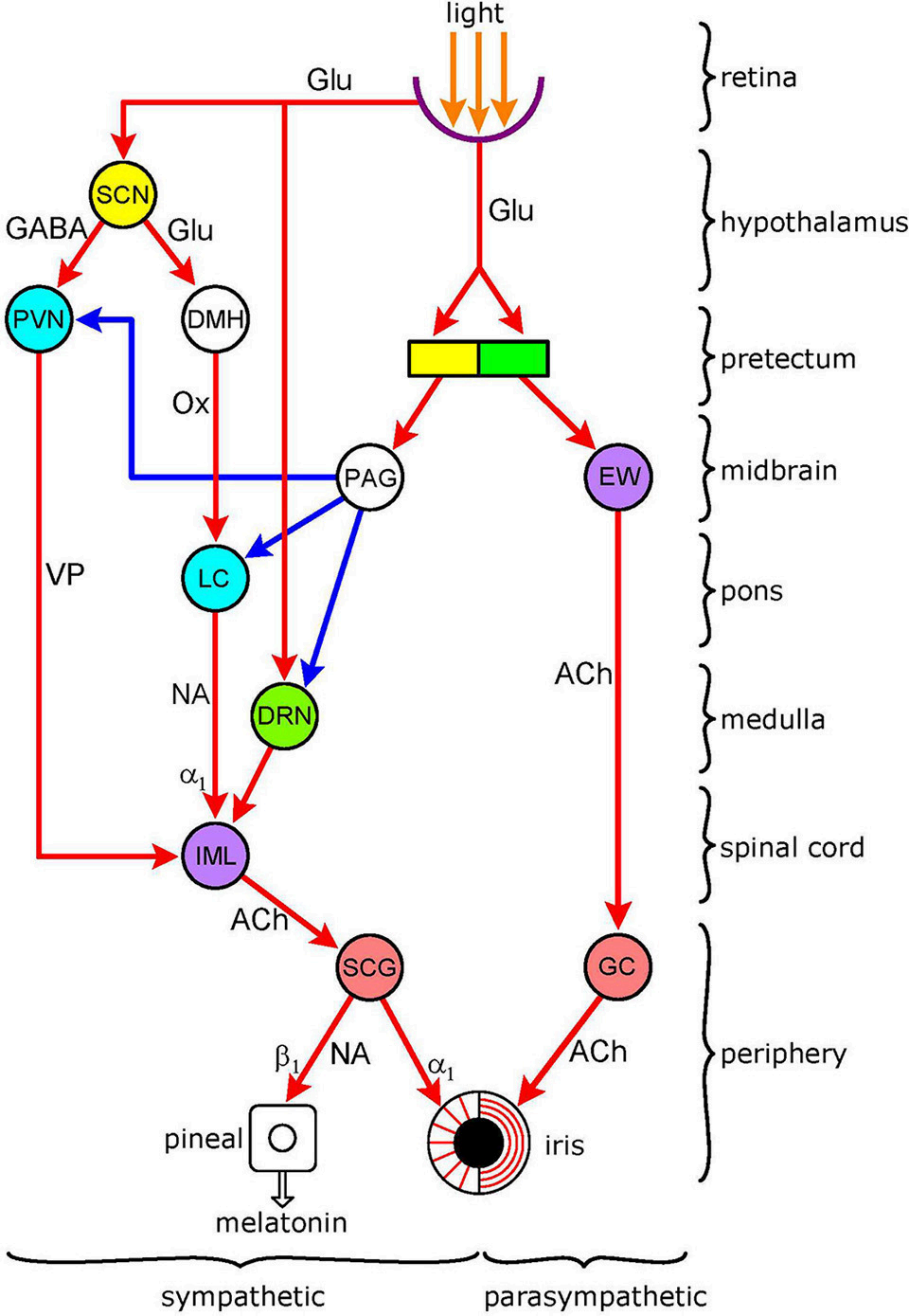
Functional organization of autonomic pathways controlling the pupil. The encircled areas represent nuclei and ganglia. *Retinoreceptive light-sensitive relay nuclei* (yellow): SCN (suprachiasmatic nucleus); pretectum. *Retinoreceptice light-sensitive premotor autonomic nuclei* (green): parasympathetic–OPN (olivary pretectal nucleus); sympathetic–DRN (dorsal raphe nucleus). *Premotor autonomic nuclei* (blue): PVN (paraventricular nucleus); LC (locus coeruleus). *Preganglionic nuclei* (purple): parasympathetic–EW (Edinger-Westphal nucleus); sympathetic–IML (intermedio-lateral column). *Integrative relay nuclei* (white): DMH (dorso-medial hypothalamus); PAG (periaqueductal gray. *Autonomic ganglia* (pink): sympathetic–SCG (superior cervical ganglion); parasympathetic–GC (ganglion ciliare). *Connections* are shown by arrows: red-excitatory; blue-inhibitory. *Neurotransmitters*: Glu (glutamate); GABA (y-amino-butyric acid); Ox (orexin); VP (vasopressin); NA (noradrenaline); ACh (acetylcholine). *Adrenoceptors* (postsynaptic): α_1_ (excitatory); β_1_ (excitatory). There are 5 *light-modulated autonomic pathways*: (1) parasympathetic (light-stimulated): OPN → EW → GC → sphincter pupillae muscle; (2) sympathetic (light-inhibited): pretectum → PAG → sympathetic premotor nuclei (PVN, LC, DR) → IML → SCG → dilator pupillae muscle; (3) sympathetic (light-inhibited): SCN → PVN → IML → SCG → dilator pupillae muscle; (4) sympathetic (light-stimulated): SCN → DMH → LC → IML → SCG → dilator pupillae muscle; (5) sympathetic (light-stimulated): DR → IML → SCG → dilator pupillae muscle. Please note overlap of pathway 3 with control of melatonin synthesis. See text for details.

**Figure 2:**
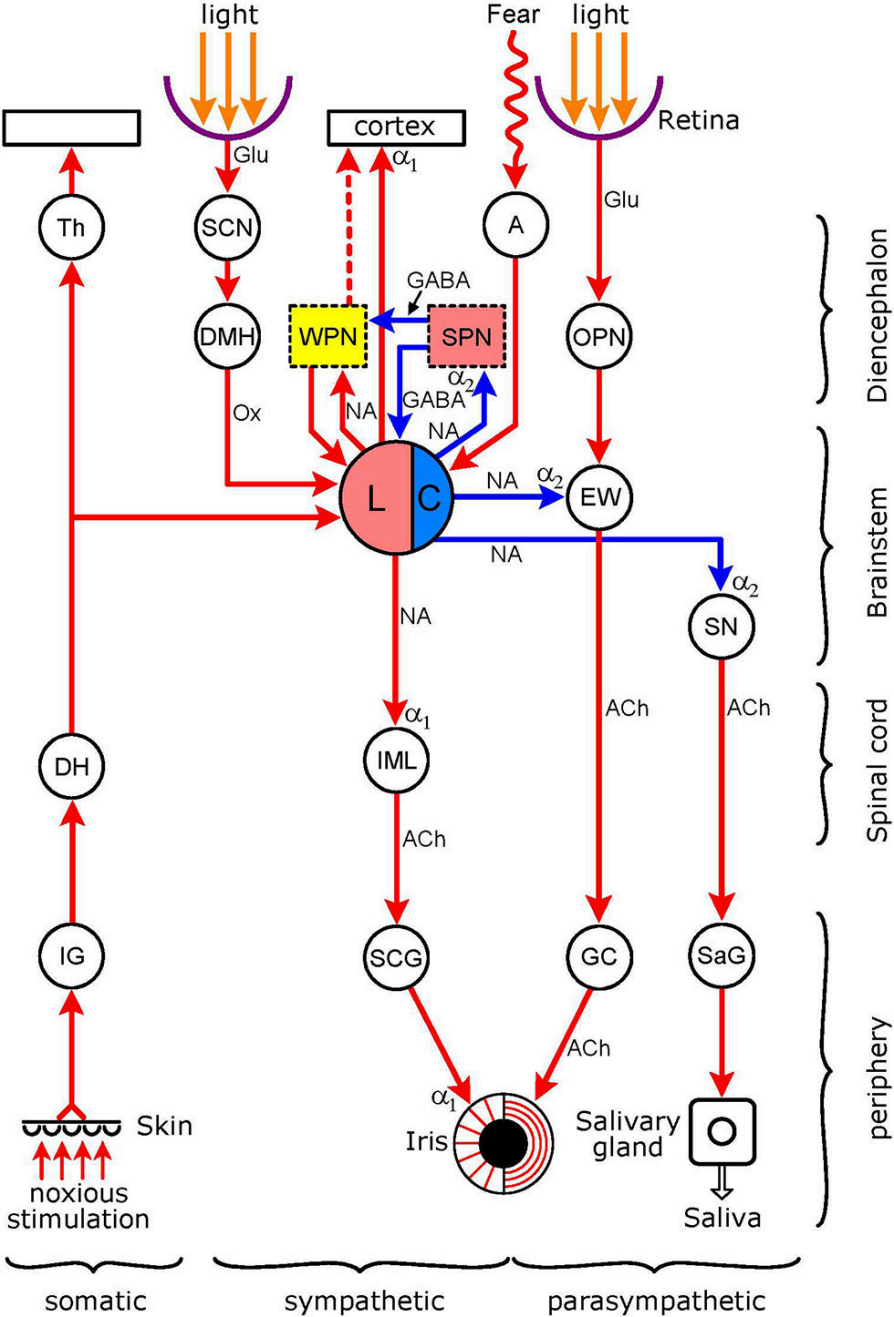
Connections of the light-stimulated noradrenergic pathway. The encircled areas represent nuclei and ganglia. *Diencephalon* Th (thalamus); SCN (suprachiasmatic nucleus of hypothalamus); DMH (dorso-medial hypothalamus). WPN (yellow): wake-promoting nuclei (basal forebrain, thalamus, pedunculopontine tegmental nucleus, tuberomamillary nucleus. ventral tegmental area, dorsal raphe nucleus); SPN (pink): sleep-promoting nuclei (ventrolateral preoptic nucleus of hypothalamus, basal forebrain). A, amygdala; OPN, olivary pretectal nucleus. *Brainstem*: LC, locus coeruleus (pink: excitatory sympathetic premotor neurones, blue: inhibitory parasympathetic premotor neurones); EW (EWN in text: Edinger-Westphal nucleus); SN, salivatory nucleus. *Spinal cord*: DH (dorsal horn); IML (intermedio-lateral column). *Peripheral ganglia*: IG (intervertebral somatosensory ganglion); SCG (superior cervical ganglion); GC (ganglion ciliare); SaG (salivatory ganglion). *Connections* are shown by arrows: red–excitatory; blue–inhibitory. *Neurotransmitters*: Glu (glutamate); GABA (y-amino-butyric acid); Ox (orexin); NA (noradrenaline); ACh (acetylcholine). *Adrenoceptors* (postsynaptic): α_1_ (excitatory); α_2_ (inhibitory). The *excitatory sympathetic premotor neurones in the LC* are stimulated by light (via the retina → SCN → DMH → LC pathway), by pain (via collaterals from the spino-thalamic tract), and via inputs from WPN during wakefulness; this would lead to an increase in sympathetic outflow to the iris (LC → IML → SCG → dilator pupillae muscle), manifesting as pupil dilation. The excitatory sympathetic premotor neurones in the LC are inhibited by SPN during sleep, leading to a reduction in sympathetic outflow to the iris, manifesting as pupil constriction. The *inhibitory parasympathetic premotor neurones in the LC* can be activated by fear and anxiety, via an input from the amygdala, leading to enhancement of the inhibition of parasympathetic preganglionic neurones in the EW (inhibition of light reflex: retina → OPN → GC → sphincter pupillae muscle pathway) and in the SN (reduction in salivary output: SN → SaG → salivary gland pathway). For WPN and SPN, see Szabadi (6). See text for details.

The basic autonomic mechanism controlling the pupil is straightforward: pupil constriction is mediated via parasympathetic activation of the circular sphincter pupillae muscle, and dilation via sympathetic activation of the radial dilator pupillae muscle (1). The autonomic pathways regulating the pupil are illustrated in Figure 1. Both the sympathetic and parasympathetic controls are organized in a hierarchical fashion, in an ascending order from the periphery, to the spinal cord, brainstem, hypothalamus, and finally cerebral cortex (not shown). The autonomic output pathways have the general structure of autonomic efferents: two serially connected neurones synapsing in autonomic ganglia. Both the ganglia and the pre-ganglionic neurones projecting to them are well defined for pupillary control. Sympathetic preganglionic neurones in the “ciliospinal center” in the intermedio-lateral nuclear column (IML) of the cervico-thoracic spinal cord [segments C8-T2] project to the superior cervical ganglion (SCG), and parasympathetic preganglionic neurones in the Edinger-Westphal nucleus (EWN) of the midbrain project to the ganglion ciliare (GC). It should be noted that the EWN is not a homogenous structure: apart from preganglionic parasympathetic cholinergic neurones (EWpg) innervating the GC, there is also a population of centrally-projecting urocortin-containing neurones (EWcp) in the nucleus (2). Autonomic outflow to the iris is modulated by central autonomic pathways projecting to the preganglionic neurones via premotor autonomic neurones. Sympathetic promotor nuclei are the paraventricular nucleus (PVN) in the hypothalamus and the locus coeruleus (LC) and dorsal raphe nucleus (DRN) in the brainstem; parasympathetic premotor nuclei are the olivary pretectal nucleus (OPN) and the LC (see Figure 2). Some of the premotor nuclei are light-sensitive, either directly (DRN, OPN) or indirectly (PVN, LC), receiving luminance information from light-sensitive areas (see sections Pretectum/Periaqueductal Gray Pathway, Suprachiasmatic Nucleus/Paraventricular Nucleus Pathway, Dorsomedial Hypothalamus, and Figure 2, below).

In order to unravel the complexity of central autonomic regulation, it has been suggested to consider central autonomic control in terms of the functional organization of autonomic pathways (3–5, 7). Organizing principles have been suggested, such as target (5) or sensory input (4). Examples of functional organization have been presented (3, 7). However, the autonomic control of the pupil receives only patchy treatment in these papers.

As the fundamental function of the autonomic innervation of the pupil is to transmit the effect of light, it is proposed that the effect of light be used as the organizational principle in the case of pupil-controlling autonomic pathways. While light has a robust stimulatory effect on parasympathetic outflow, it has a dual (inhibitory/stimulatory) effect on sympathetic outflow. Thus the parasympathetic output is controlled by a light-stimulated pathway, whereas the sympathetic outflow is controlled by separate light-inhibited and light-stimulated pathways. The light-inhibited sympathetic pathway is yoked to the light-stimulated parasympathetic pathway mediating the pupillary light reflex: as the pupil is constricted by stimulation of the parasympathetic pathway, sympathetically mediated pupil dilation is withdrawn (8). The activity of the light-stimulated sympathetic pathways is less obvious since it is masked by sympatho-inhibition evoked by light. This masked effect can be revealed by pharmacological means, as discussed below (see section Noradrenergic Pathway). These pathways operate via more than one sympathetic premotor nucleus, and play an important role in mediating the effects of a number of physiological (arousal, pain, high temperature) and psychological (attention, mood, anxiety) variables on the pupil.

## Light-Inhibited Sympathetic Pathways

Early work has shown that light inhibits neuronal activity in efferent peripheral sympathetic fibers, recorded from both preganglionic (“sympathetic nerves”) (9–11) and postganglionic (long ciliary nerves) (12, 13) fibers, in cats. The reduction in discharge is linearly related to the intensity of the light stimulus (10). The sympathetic pathways conveying the effect of light originate from retina-receptive light-sensitive sites in the brain that project to the ciliospinal center (10, 12) Two possible candidates for the sites of origin of light-inhibited sympathetic pathways are the pretectum and the suprachiasmatic nucleus (SCN) of the hypothalamus. These pathways are displayed in Figure 1 (for detailed description of the figure, see section Introduction, above).

### Pretectum/Periaqueductal Gray Pathway

Early work by Okada et al. (13) provided experimental evidence in support of the hypothesis that the light-inhibited sympathetic pathway to the pupil, like the light reflex pathway (14), might originate from the pretectum. These authors introduced serial brainstem lesions to disrupt this putative pathway in an anesthetized cat preparation. On the basis of the effects of the lesions on light-inhibited sympathetic activity in long ciliary nerves, they concluded that there was a neural connection running from the pretectum to the cervical sympathetic. As the output from the pretectum to the parasympathetic preganglionic neurones in the Edinger-Westphal nucleus is excitatory, it remains to be explained how an inhibitory sympathetic pathway originates from the same area.

Two groups of light-sensitive neurones have been identified in the pretectum: one group in the olivary pretectal nucleus (“luminance detectors”) that is stimulated by light, and another one (“darkness detectors”) in the posterior pretectal nucleus that is inhibited by light (15). An attractive possibility may be that the light-stimulated parasympathetic and light-inhibited sympathetic pathways originate from these two different populations of light-sensitive pretectal neurones: the parasympathetic pathway from the luminance detectors, and the sympathetic pathway from the darkness detectors. However, there is no evidence to support this hypothesis.

More recent experimental evidence supports the existence of a neural link between the pretectal area and sympathetic premotor neurones. A direct link has been identified between the anterior pretectal nucleus and the rostral ventrolateral medulla (16), a major location of sympathetic premotor neurones in the brainstem (17). However, there is no evidence that the anterior pretectal nucleus is light-sensitive, and the rostral ventrolateral medulla is involved mainly in cardiovascular regulation (17). Therefore, it is likely that the inhibitory effect of light on sympathetic outflow to the iris is transmitted indirectly via the periaqueductal gray matter (PAG) of the midbrain. It has been shown that a projection from the OPN reaches sympathetic preganglionic neurones in the upper thoracic spinal cord and postganglionic neurones in the SCG via the PAG (18).

The PAG functions as an integrative relay nucleus (19). Sympathetic premotor neurones innervated by the PAG include the C1 (adrenergic) neurones in the rostral ventrolateral medulla, noradrenergic neurones in the A5 and A6 (locus coeruleus) nuclei, serotonergic neurones in the medullary raphe nuclei, and the PVN (20). Interestingly, the synapses of the PAG neurones on sympathetic premotor neurones have the morphological features of inhibitory synapses, and, therefore, it is assumed that the PAG may exert an inhibitory influence on the innervated postsynaptic cells (18, 21, 22).

Therefore, it is likely that, in the case of this light-inhibited sympathetic pathway, an excitatory output from the light-sensitive cells of the pretectum is converted into an inhibitory signal by the PAG (Figure 1).

### Suprachiasmatic Nucleus/Paraventricular Nucleus Pathway

The PVN has been identified as a major sympathetic premotor nucleus (23), and its roles in the regulation of cardiac (24, 25) renal, (24) and liver functions, and melatonin synthesis (26, 27) are well documented in experiments conducted in rodents. It has been shown that the PVN exerts an excitatory effect on sympathetic preganglionic neurones via the neuropeptides vasopressin and oxytocin (28, 29). The PVN receives an input from the retina-recipient light-sensitive cells of the SCN of the hypothalamus, the “biological clock of the brain.” It has been shown that, via this connection, light exerts a marked circadian influence on some sympathetic functions controlled by the PVN, such as melatonin synthesis (27) and glucose metabolism (30).

There is an overlap between the sympathetic controls of melatonin synthesis by the pineal gland and that of pupil dilation by the dilator muscle of the iris. In the case of both functions, the preganglionic neurones are located in the C8-T2 segments of the IML, and project to the SCG. This overlap is highlighted by a clinical observation: bilateral oculo-sympathetic paresis (Horner's syndrome) resulting from injury to the lower cervical/upper thoracic spinal cord leads to the cessation of nocturnal melatonin secretion (31).

The neuronal pathway controlling melatonin synthesis is well established: it runs from the SCN to the PVN, that projects to the preganglionic neurones in the IML (26) (Figure 1). Light exerts an inhibitory influence on melatonin synthesis via stimulation of an inhibitory GABAergic output from the SCN to the PVN. Two GABAergic inhibitory mechanisms have been identified in the SCN: (1) a time-of-day-dependent circadian mechanism that switches off the premotor neurones in the PVN during daytime, leading to the cessation of melatonin synthesis for the day phase of the day/night cycle; and (2) a light-activated inhibitory mechanism that becomes operational at night-time, when melatonin synthesis is released from its circadian inhibition, leading to acute suppression of melatonin synthesis (32).

Premotor neurones in the PVN involved in pupillary control are likely to be separate from those controlling melatonin synthesis since there is no evidence that pupil control is subject to the same circadian regulation as melatonin synthesis. However, these neurones, like those controlling melatonin synthesis, may also be susceptible to the direct inhibitory effect of light relayed via the SCN. Thus the SCN may give rise to a light-inhibited sympathetic pathway controlling pupillary function (Figure 1). On the other hand, the light-inhibited projection from the pretectum controlling pupil dilation (see Pretectum/Periaqueductal Gray Pathway), via inhibiting PVN activity, may contribute to the suppression of melatonin synthesis by light.

## Light-Stimulated Sympathetic Pathways

It is well established that light constricts the pupil by stimulating the parasympathetic output to the constrictor pupillae muscle via the light reflex pathway, and that pupil constriction is facilitated by the concurrent inhibition of the sympathetic output to the dilator pupillae muscle (1). Indeed, when recording from pre- or post-ganglionic sympathetic fibers innervating the iris, an inhibition of impulse flow in response to light has been detected (see section Light-Inhibited Sympathetic Pathways). Therefore, any increase in impulse flow in response to light would be masked by the dominant inhibitory effect. A stimulatory effect of light on the sympathetic control of the pupil, using pupil dilation as its corollary, could be unmasked by drugs modulating the activity of potential light-stimulated sympathetic pathways (see Pharmacological Unmasking of Light-Evoked Latent Pupil Dilation).

### Noradrenergic Pathway

This pathway, with some of its connections, is shown in Figure 2. This Figure, like Figure 1, displays the basic autonomic control of the pupil, the sympathetic output projecting to the dilator pupillae muscle and the parasympathetic output to the sphincter pupillae muscle of the iris. The figure also shows the light reflex pathway (retina → OPN → EWN → GC → sphincter pupillae muscle). The hub of the noradrenergic pupil-control pathway is the LC. The LC functions as both a sympathetic and parasympathetic premotor nucleus. Anatomical studies in rats have shown that the LC (A6 noradrenergic nucleus), together with the A5 and A7 noradrenergic nuclei, projects to the spinal cord where noradrenergic axon terminals reach sympathetic preganglionic neurones [(33, 34), see also Figure 4 in (35)]. Furthermore, this projection is likely to be excitatory via postsynaptic α_1_-adrenoceptors (36). The LC also projects to parasympathetic preganglionic neurones in the EWN (see Outputs) and the salivatory nuclei (SN) (37, 38). The LC exerts an inhibitory influence on preganglionic parasympathetic neurones via the stimulation of α_2_-adrenoceptors (39, 40). The LC sends a rich ascending excitatory projection to the cerebral cortex, and functions as a major wake-promoting nucleus (41–44). Inputs to the LC include an indirect excitatory connection from the retina-recipient light-sensitive neurones of the SCN via the dorsomedial hypothalamus (DMH) (see Dorsomedial hypothalamus), excitatory inputs from the wake-promoting neurones (WPN) of the sleep-arousal network and inhibitory inputs from sleep-promoting neurones (SPN) of the sleep-arousal network (see Association With Sleep/Arousal Network), an excitatory input from the amygdala to parasympathetic premotor neurones (see Amygdala), and an excitatory input from the spinothalamic pathway conveying pain sensation (see Collaterals From Spinothalamic Tract).

The anatomical basis for the classification of the noradrenergic pathway as a light-sensitive pathway is an indirect connection from the retina to the LC via the SCN and DMH, identified by Aston-Jones and his colleagues [(45, 46); for a recent review see (47)]. There is evidence that light activates the LC both in humans and diurnal animals. It has been shown in humans by fMRI that light causes activation in a brain area corresponding to the LC (48), and in Nile grass rats, a species of diurnal rodents, increases the expression of cFOS, a marker of neuronal activity (49), both in the SCN and the LC (50). Furthermore, light exerts effects consistent with LC activation. It increases the level of arousal in both humans (51, 52) and diurnal animals (50, 53), and enhances sympathetic activity in both humans (51, 54), and animals, such as mice (55, 56).

The involvement of the LC in pupil control is well established. When recording simultaneously the firing rate of LC neurones and the diameter of the pupil in monkeys, a close parallelism could be observed between fluctuations in firing rate and pupil diameter (57). More recently, it has been reported that electrical microstimulation of the LC in monkeys (58) and rodents (59, 60) leads to pupil dilation. In humans, it has been shown with fMRI that pupil dilation responses to psychological stimuli correlate with activation in a brain area overlapping with the LC (61, 62).

#### Pharmacological Unmasking of Light-Evoked Latent Pupil Dilation

As light apparently constricts the pupil, any latent dilation of the pupil resulting from sympathetic activation via the noradrenergic pathway would be masked by pupil constriction resulting from sympathetic inhibition via the pretectum/PAG and SCN/PVN pathways, and parasympathetic stimulation via the OPN/EWN/GC pathway. The latent pupil dilation evoked by light can be unmasked by drugs that modulate the activity of the noradrenergic pathway via LC activity. The activity of central noradrenergic neurones is regulated by inhibitory α_2_-adrenoceptors on the noradrenergic neurones themselves (“autoreceptors”): α_2_-adrenoceptor agonists (e.g., clonidine) “switch off” the activity of these neurones, whereas α_2_-adrenoceptor antagonists (e.g., yohimbine) enhance it [see (44, 63)] (see α2-Adrenoceptors Associated With Premotor Autonomic Neurones).

Clonidine, by switching off the LC neurones in the noradrenergic sympatho-excitatory pathway to the pupil, causes pupil constriction in man. Interestingly, this effect is light-dependent: the reduction in pupil diameter in response to clonidine is greater in light than in darkness (64). This is likely to reflect a “baseline effect” (65): in darkness the baseline (i.e., sympathetic activity) is low, leading to an attenuated response to the sympatholytic drug clonidine; increasing latent sympathetic activity by light, and thus elevating the baseline, would enhance the response to the sympatholytic drug. A corollary to the potentiation of the miotic effect of clonidine by light is the potentiation of light-evoked pupil constriction by clonidine. When pupil diameter in light is used as a measure of pupil constriction, the light stimulus intensity/pupil diameter curve (pupil diameter plotted against logarithm of light intensity) is shifted to the left (66, 67). Thus the same light intensity evokes a larger response, or the same response is evoked by a lower intensity stimulus, indicative of potentiation. On the other hand, the α_2_-adrenoceptor antagonist yohimbine has the opposite effect: it shifts the light intensity/pupil diameter curve to the right, consistent with antagonism. Furthermore, when applied together, there is evidence of mutual antagonism between the effects of clonidine and yohimbine (66).

An alternative explanation for the light-dependent effect of clonidine may be that it is due to attenuation of the noradrenergic inhibition of the EWN, leading to potentiation of the light reflex (63, 68). However, potentiation of the light reflex response by clonidine is reported only rarely (see Pupillary Effects of Noradrenergic Drugs), and usually it cannot be observed at a time when there is evidence of the potentiation of light-evoked pupil constriction (67). Therefore, although occasionally there may be a parasympathetic contribution to the potentiation of light-evoked pupil constriction by clonidine, it is likely to be largely due to sympathetic inhibition.

Drugs indirectly modulating LC activity also have effects consistent with the unmasking of latent pupil dilation. The LC is activated by inputs from wake-promoting nuclei of the sleep/arousal network, such as the dopaminergic ventral tegmental area (VTA) of the midbrain (69, 70); and the histaminergic tuberomamillary nucleus (TMN) of the hypothalamus (71, 72) [for reviews, see (6, 44)]. The stimulant drug modafinil, by blocking the reuptake of dopamine at excitatory dopaminergic synapses on LC neurones (73), increases LC activity, and thus also the latent mydriatic effect of light. Indeed, modafinil shifts the light intensity/pupil diameter curve to the right, consistent with antagonism of light-evoked pupil constriction (67). Histamine, the excitatory neurotransmitter of wake-promoting tuberomamillary neurones, excites LC neurones via stimulation of H_1_ histamine receptors (6), and this excitation is blocked by H_1_ receptor antagonists (72). The H_1_ receptor antagonists would decrease LC activity and thus potentiate latent pupil dilation. Indeed, diphenhydramine, a H_1_ histamine receptor antagonist, has been shown to potentiate light-evoked pupil constriction (74). There is also evidence of antagonism between the effects of drugs that potentiate and antagonize light-evoked pupil constriction: the effect of modafinil is antagonized by clonidine (67), and the effect of diphenhydramine is antagonized by modafinil (74).

In conclusion, drugs modifying LC activity reveal the operation of a latent mydriatic effect of light that acts to attenuate light-evoked pupil constriction.

#### Functional Organization of Noradrenergic Premotor Autonomic Neurones in the Locus Coeruleus

Central noradrenergic neurones are dual function neurones: by stimulating both postsynaptic excitatory α_1_-adrenoceptors and inhibitory α_2_-adreneceptors at their postsynaptic projection targets, they can mediate both excitatory and inhibitory effects (44). This feature of the individual neurones underlies the dual function of the LC as a premotor autonomic nucleus. The LC contains both sympathetic and parasympathetic premotor neurones. The sympathetic premotor neurones send excitatory projections to the IML where they stimulate α_1_-adrenoceptors on sympathetic preganglionic neurones (34, 36), while the parasympathetic premotor neurones project to inhibitory preganglionic neurones in the EWN (see Outputs) and salivatory nuclei (38) where they stimulate α_2_-adrenoceptors (39, 40). For further details, see Noradrenergic Pathway, above, and for reviews, see 40, 41, 43. Although individual central noradrenergic neurones may have a dual excitatory/inhibitory role, projecting to several targets where they can stimulate either excitatory or inhibitory adrenoceptors, it is likely that the preautonomic neurones in the LC segregate into separate populations of excitatory sympathetic and inhibitory parasympathetic premotor neurones. These two populations are defined not only by their separate outputs but also by their separate inputs and their distinct susceptibility to physiological (light, pain) and psychological (threat) variables (41–43). Interestingly, recently two subpopulations of LC neurones have been identified on a genetic/developmental basis (75); however, there is no evidence to date whether these separate populations correspond to sympathetic and parasympathetic premotor neurones.

#### α_2_-Adrenoceptors Associated With Premotor Autonomic Neurones

Inhibitory α_2_-adrenoceptors are located at two sites: on the noradrenergic neurones themselves (“autoreceptors”) (76, 77) and on the innervated target cells (neurone, glia cell or smooth muscle of blood vessels: postsynaptic receptors) (78, 79) [for reviews, see (44, 63, 80, 81)]. The inhibitory autoreceptors operate a negative feedback mechanism dampening the activity of the noradrenergic neurone. Somatodendritic autoreceptors, stimulated by noradrenaline released from dendrites and/or recurrent axon collaterals synapsing with the cell body/dendrites, attenuate the firing of the neurone (82), whereas presynaptic receptors on nerve terminals reduce the release of the neurotransmittter (83). Stimulation of postsynaptic α_2_-adrenoceptors initiates inhibition of the cell receiving noradrenergic innervation.

There are three populations of α_2_-adrenoceptor in/or associated with the preautonomic LC: autoreceptors on (1) sympathetic, and (2) parasympathetic premotor neurones, and (3) postsynaptic receptors innervated by parasympathetic premotor neurones. Drugs interacting with α_2_-adrenoceptors can have differential effects on the three receptor populations, due to their differential sensitivities. Interestingly, there is a species difference in the sensitivities of the three receptor populations: diurnal and nocturnal animals are affected differently by α_2_-adrenoceptor agonists and antagonists.

**Figure 3:**
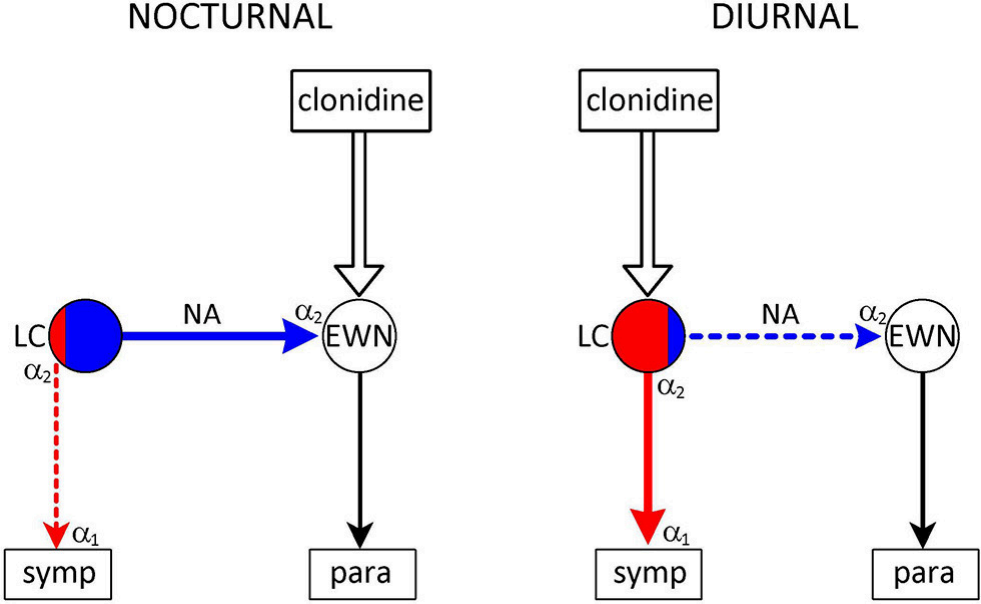
Species-specific effect of the α_2_-adrenoceptor agonist clonidine on the pupil. *Nuclei*: LC–locus coeruleus (red area: sympathetic premotor neurones; blue area: parasympathetic premotor neurones); EWN, Edinger-Westphal nucleus. symp: target of output from LC (sympathetic preganglionic neurones); para: target of output from EWN (ganglion ciliare). *Connections* are shown by arrows: red–excitatory; blue–inhibitory. *Adrenoceptors*: α_2_ in LC–presynaptic inhibitory autoreceptor; α_2_ in EWN–inhibitory postsynaptic receptor; α_1_ on sympathetic preganglionic neurones–excitatory postsynaptic receptor. *Nocturnal animals*: clonidine stimulates inhibitory postsynaptic receptors in the EWN–inhibition of the EWN leads to mydriasis. *Diurnal animals:* clonidine stimulates inhibitory autoreceptors on sympathetic premotor neurones in the LC–attenuation of sympathetic outflow leads to miosis. See text (α2-Adrenoceptors Associated With Premotor Autonomic Neurones) for details.

In diurnal species (man, monkey, dog), the α_2_-adrenoceptor agonist clonidine evokes miosis and sedation, consistent with a sympatholytic effect resulting from stimulation of inhibitory autoreceptors on sympathetic premotor neurones in the LC (42, 43, 63) (Figure 3). This is a selective effect: the other two populations of α_2_-adrenoceptor remain largely unaffected. This may be due partly to the greater sensitivity of autoreceptors than postsynaptic receptors (84, 85), and partly the higher activity of sympathetic, compared to parasympathetic, premotor neurones. Sympathetic premotor neurones are likely to be more active due to their preferential stimulation by light (see Dorsomedial hypothalamus), and autoreceptor activity is a function of neuronal activity (81).

In contrast to diurnal animals, clonidine causes mydriasis in nocturnal (mouse, rat) and crepuscular (cat) animals (42, 43, 63) (Figure 3). This is consistent with the selective stimulation of postsynaptic inhibitory α_2_-adrenoceptors in the EWN (40), innervated by a noradrenergic output from parasympathetic premotor neurones in the LC (see Outputs). The lack of evidence of autoreceptor stimulation in these neurones may be due to their presumed low baseline activity in the absence of stimulation by light.

#### Connections of Excitatory Sympathetic Premotor Neurones

##### Outputs

There is a robust projection from the LC to the spinal cord (coeruleo-spinal pathway), demonstrated in the rat (86). This pathway innervates all three contingents of spinal neurones: autonomic preganglionic neurones in the IML (87), sensory neurones in the dorsal horn (88), and motor neurones in ventral horn (89). The ciliospinal center receives its noradrenergic innervation via this pathway (41, 44).

Excitatory LC neurones also operate in the sleep/arousal network: they project to other wake-promoting neurones and the cerebral cortex (see Association With Sleep/Arousal Network).

##### Inputs

*Latero-posterior hypothalamus*. The orexinergic neurones in the lateral hypothalamic area/perifornical area (LHA/PFA) play an important role in the control of both arousal (6) and autonomic regulation (90): they mediate wake-promoting (91) and sympatho-excitatory (92) effects. The sympatho-excitatory effects of these neurones are mediated either directly by their projections to sympathetic preganglionic neurones (92), or indirectly via projections to sympathetic premotor neurones in the rostral ventrolateral medulla (RVLM) (90) and PVN (93). By projections to sympathetic premotor neurones in the LC (94), the orexinergic neurones of the LHA/ PFA stimulate sympathetic outflow to the iris. Interestingly, the hypothalamus, and in particular the latero-posterior hypothalamus containing the orexinergic neurones, has been implicated for a long time in the sympathetic control of the pupil (78, 95). Pupil dilatory responses reported in response to the electrical stimulation of the latero-posterior hypothalamus [see Table 6–19 in (96)] are likely to have been due to activation of orexinergic neurones projecting to the LC. Furthermore, the “tonic inhibition” of the EWN observed following hypothalamic stimulation may reflect the activation of parasympathetic premotor neurones in the LC in response to stimulation of the orexinergic input (see Connections of Inhibitory Parasympathetic Premotor Neurones).

There is also clinical evidence highlighting the importance of the lateral hypothalamus in the sympathetic control of the pupil. It is recognized that lesions of the postero-lateral hypothalamus can cause central type ipsilateral Horner's syndrome, characterized by miosis, blepharoptosis, and facial anhidrosis. This reflects the loss of the sympathetic output from the hypothalamus channeled through a descending pathway, via the SCG, to the iris (97).

*Dorsomedial hypothalamus*. The DMH projects to the LC (45) that sends excitatory outputs to the IML, wake-promoting neurones in the sleep/arousal network, and the cerebral cortex (see Noradrenergic Pathway, para 1). Via these connections light increases both the level of arousal and sympathetic activity in diurnal animals, including man (50, 51, 98, 99). This direct effect of light is separate from the effect of light on the entrainment of circadian rhythmicity to the day/night cycle, and is often referred to as “masking,” since it used to be regarded as a side-effect in the study of circadian regulation (50).

**Figure 4:**
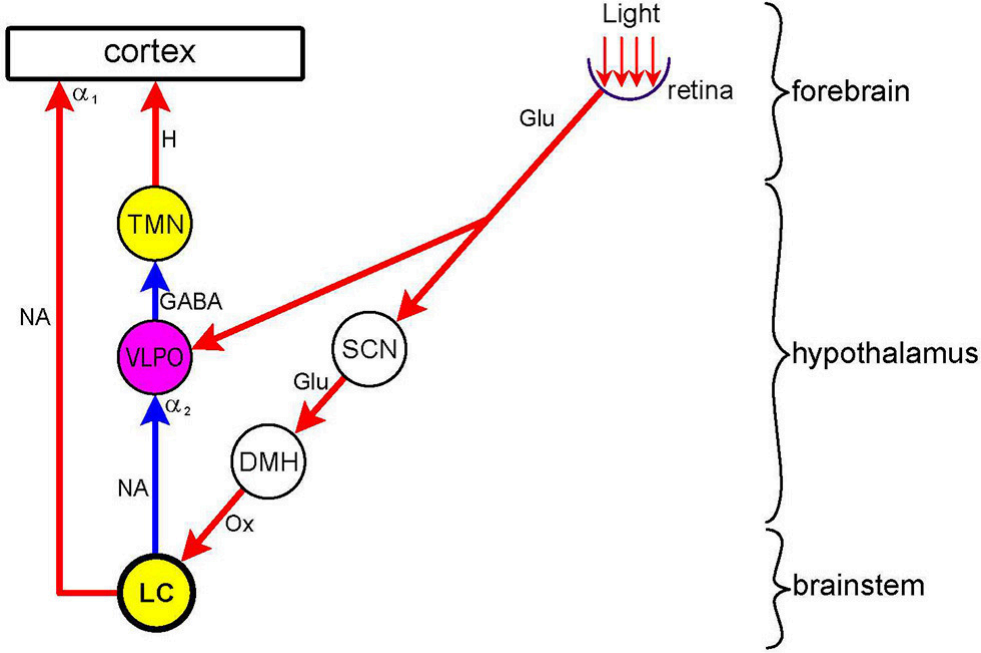
Dual effect of light on arousal *Nuclei*: wake-promoting (yellow)–LC (locus coeruleus); TMN (tuberomamillary nucleus); sleep-promoting (purple)–VLPO (ventrolateral preoptic nucleus; relay (white)–SCN (suprachiasmatic nucleus); DMH (dorsomedial hypothalamus. *Connections* are shown by arrows: red–excitatory; blue–inhibitory. *Neurotransmitters*: Glu (glutamate); GABA (y-amino-butyric acid); Ox (orexin); H (histamine); NA (noradrenaline). *Adrenoceptors* (postsynaptic): α_1_ (excitatory), α_2_ (inhibitory). Light exerts a sleep-promoting effect by directly stimulating the sleep-promoting nucleus VLPO. This effect is largely mediated by inhibition of the wake-promoting nucleus TMN. Light also exerts a wake-promoting effect by indirectly stimulating, via the SCN and DMH, the wake-promoting nucleus LC. This effect is mediated by the direct stimulation of the cerebral cortex and the inhibition of the VLPO, thereby disinhibiting the wake-promoting TMN. In nocturnal animals the sleep-promoting, and in diurnal animals the wake-promoting, effect of light predominates. See text (Dorsomedial Hypothalamus) for details.

In contrast to diurnal animals, light is sleep-promoting (“somnogenic”), and darkness is wake-promoting in nocturnal animals, including rodents used in laboratory research (50, 100–102). Interestingly, in nocturnal animals the wake-promoting orexinergic neurones in the hypothalamus are activated by darkness (103), whereas in diurnal animals they are activated by light (104). The dual effect of light on arousal is likely to reflect the opposite effects of light on two hypothalamic nuclei: stimulation of the SCN leads to an alerting effect via the DMH and LC, whereas the stimulation of the ventrolateral preoptic nucleus (VLPO), a major sleep-promoting nucleus, leads to a sedative effect (43, 44, 102, 105) (Figure 4). In diurnal animals light would activate predominantly the SCN, while in nocturnal animals the predominant effect of light would be the activation of the VLPO. It has been suggested that the basis for the “temporal niche” (i.e., diurnality or nocturnality) may lie in the retina (106, 107). This suggestion has received experimental support recently (105). It has been shown that blue light has an alerting and green light a sedative effect. Therefore diurnal animals may show a higher sensitivity to blue in the spectrum, and retinal ganglion cells stimulated by blue light may project preferentially to the SCN. On the other hand, nocturnal animals' retinae may be more sensitive to green in the spectrum, and retinal ganglion cells stimulated by green light, may project preferentially to the VLPO (53, 105, 108).

In diurnal animals, luminance information is channeled via the SCN → DMH route to the LC leading to activation of not only wake-promoting but also sympathetic premotor neurones. The latter activation leads to an increase in sympathetic outflow in general, including cardiovascular activity, and increased sympathetic stimulation of the iris manifesting as pupil dilation [see Figure 9 in (44)]. Activation of the wake-promoting neurones in the LC leads to activation of the cerebral cortex, both directly via the coeruleo-cortical pathway (41, 44), and indirectly via shifting the overall activity of the subcortical sleep-arousal network in the direction of wake-promotion (6). Interestingly, cortical activation may involve areas associated with processing non-luminance-related information, such as cognitive load. As these cortical areas are known to project to the LC (58, 59, 109), their activation would provide reinforcing positive feedback to luminance-evoked LC activation.

**Figure 5:**
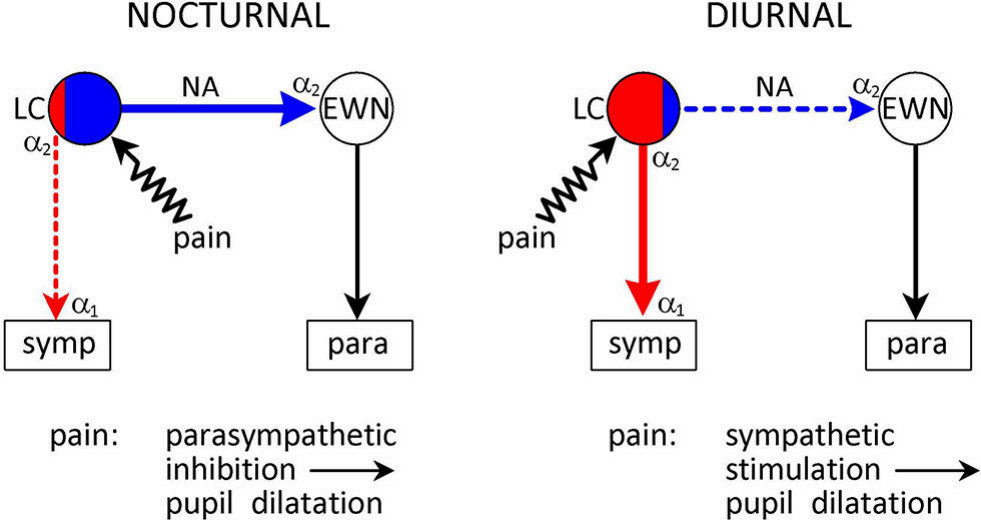
Species-specific effects of noxious stimulation on the pupil. Conventions are as in Figure 3. Pain signals arising from noxious (painful) stimulation are conveyed to the LC via collaterals from the spinothalamic tract. Pain signals evoke pupil dilation (“reflex dilation”) in both nocturnal and diurnal animals. However, the mechanisms are different. In nocturnal animals pain signals stimulate parasympathetic premotor neurones in the LC that project to the EWN to stimulate inhibitory α_2_-adrenoceptors, mediating a parasympatholytic effect. On the other hand, in diurnal animals pain signals stimulate sympathetic premotor neurones in the LC, leading to a sympatho-excitatory effect. See text (Collaterals From Spinothalamic Tract) for details.

*Collaterals from spinothalamic tract*. Pain signals are carried, via the somatosensory nucleus of the thalamus, to the somatosensory area of the cerebral cortex, by the spinothalamic (110, 111) and trigemino-thalamic (112) pathways (for review, see 42). Both pathways send collaterals to the LC, as demonstrated in the cat and monkey (113) (Figure 2). Pain signals increase LC activity, as shown in the rat by recording the electrical discharge of LC neurones (114, 115), expression of cFos (116), and noradrenaline release (117). Noxious stimulation also leads to pupil dilation, referred to as “reflex dilation.” There is evidence that reflex dilation in humans and other diurnal species (e.g., rabbit) is related to sympathetic activation, suggesting that the collaterals from pain pathways synapse with excitatory sympathetic premotor neurones in the LC (Figure 5).

In humans, reflex dilation can be evoked by noxious cold (plunging one hand into ice-cold water: “cold pressor test”) (118, 119), or electric shock (120, 121). Pupil dilation evoked by acute pain is a pure sympathetic response: the amplitude of the light reflex response, an index of parasympathetic activity, is unaffected (118, 119, 122) Reflex dilation can be antagonized by sympatholytics: α_1_-adrenoceptor antagonists (e.g., dapiprazole) applied topically to the cornea (119, 121), or α_2_-adrenoceptor agonists (e.g., dexmedetomidine) applied systemically (120).

In rabbits, another diurnal species, the noxious stimulus used was electrical stimulation of the sciatic nerve (123). Reflex dilation was antagonized by α_1_-adrenoceptor antagonists (phentolamine, phenoxybenzamine, and prazosin), and potentiated by the α_2_-adrenoceptor antagonist RS79948, administered systemically (123). The α_1_-adrenoceptor antagonists may have blocked α_1_-adrenoceptors at two sites (IML and iris) in the noradrenergic sympathetic pathway originating from the LC, whereas the α_2_-adrenoceptor antagonist may have antagonized inhibitory autoreceptors in the LC. The α_1_- and α_2_-adrenoceptor antagonists failed to affect reflex dilation in the eye whose sympathetic input had been sectioned (124). Therefore, reflex dilation in the rabbit, like in man, is likely to be mediated by sympathetic excitation originating in sympathetic premotor neurones in the LC.

In contrast to diurnal species, in nocturnal animals, reflex dilation seems to reflect parasympathetic inhibition rather than sympathetic stimulation (Figure 5). Reflex dilation in cats (crepuscular animals) and rats was studied extensively by Michael Koss and his colleagues in the 1980s (40, 78, 125, 126). They found that pupil dilation evoked by painful electrical stimulation of the sciatic nerve was not affected by sympathectomy, was antagonized by monoamine depletion by reserpine or α-methyl-para-tyrosine and α_2_-adrenoceptor antagonists (e.g., yohimbine), and potentiated by α_2_-adrencoptor agonists (e.g., clonidine). They concluded that noxious stimulation in cats and rats activated a noradrenergic pathway inhibiting the EWN, leading to a reduction in parasympathetic outflow, appearing as pupil dilation (40) (see Collaterals From Spinothalamic Tract).

The differential effect of noxious stimulation on the noradrenergic control of pupil function in diurnal and nocturnal animals suggests that while in diurnal animals pain signals may preferentially activate excitatory sympathetic premotor neurones in the LC, in nocturnal animals they may activate mainly inhibitory parasympathetic premotor neurones projecting to the EWN (Figure 5).

#### Connections of Inhibitory Parasympathetic Premotor Neurones

##### Outputs

**Figure 6:**
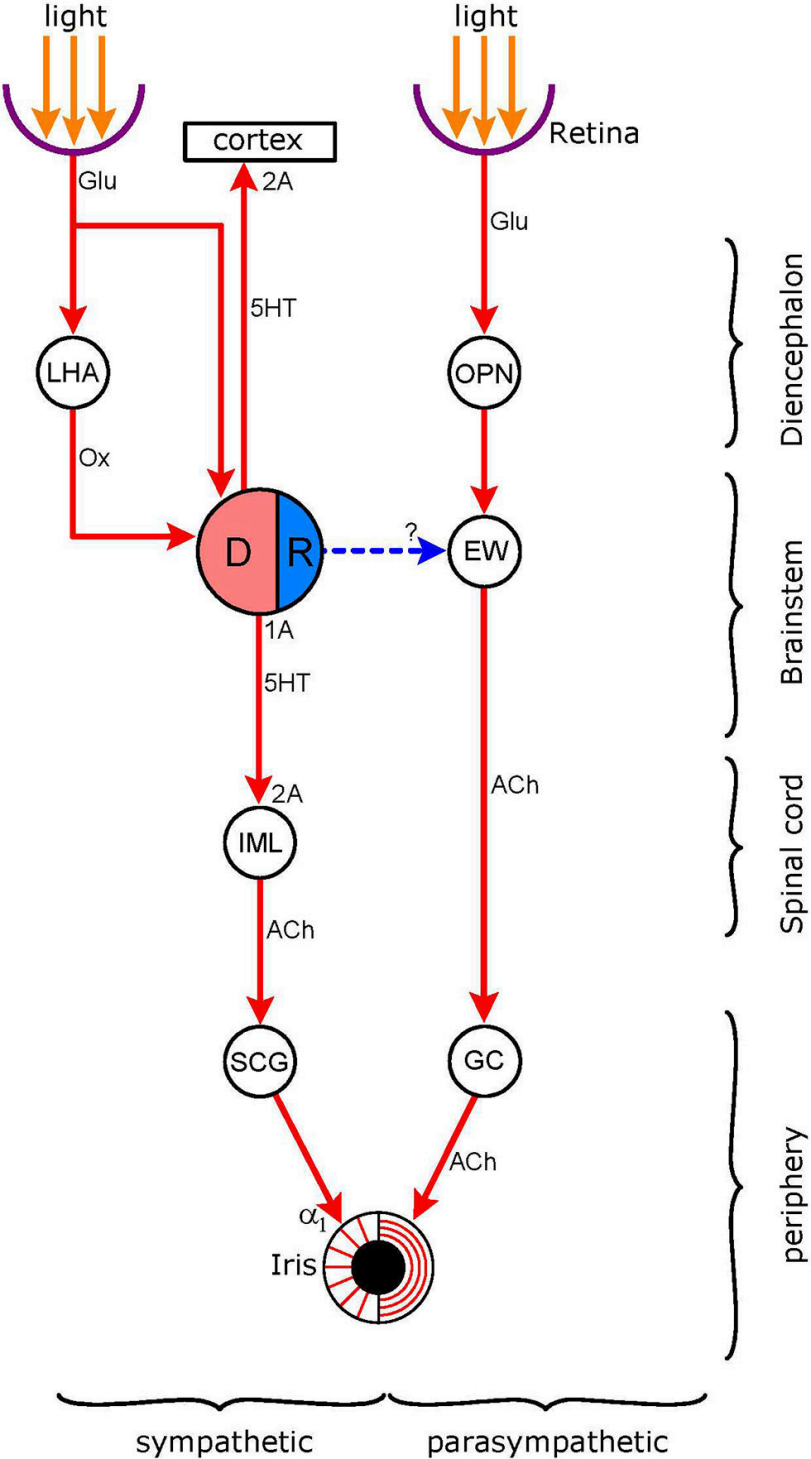
Connections of the light-stimulated serotonergic pathway. *Nuclei*: DR (DRN in text: dorsal raphe nucleus; pink: excitatory sympathetic premotor neurones, blue: inhibitory parasympathetic premotor neurones); EW (EWN in text: Edinger-Westphal nucleus); OPN (olivary pretectal nucleus); LHA (lateral hypothalamic area), IML (intermedio-lateral column of spinal cord). *Ganglia*: SCG (superior cervical ganglion), GC (ganglion ciliare). *Connections* are shown by arrows: red-excitatory; blue-inhibitory (putative). *Neurotransmitters*: Glu (glutamate); Ox (orexin), 5HT (5-hydroxytryptamine, serotonin), ACh (acetylcholine). *Receptors*: α_1_-excitatory postgsynaptic adrenoceptors; 2A–excitatory postsynaptic 5-HT_2A_ receptors; 1A–inhibitory 5-HT_1A_ autoreceptors. The DR receives both a direct and an indirect input from the retina; the indirect connection involves hypothalamic orexinergic neurones. The possibility of an inhibitory output from the DR to the EW, stimulating inhibitory postsynaptic 5-HT_1A_ receptors, has been investigated (see 5-HT1A Receptors).

It has been shown in cats and rats that there is a central noradrenergic pathway that exerts an inhibitory influence on the EWN by stimulating inhibitory α_2_-adrenoceptors (40). Moreover, it has been proposed that the inhibitory noradrenergic pathway to the EWN may originate from the LC, and that the LC could exert dual influence on pupillary activity via an excitatory output to the IML and an inhibitory output to the EWN (63) This model of the dual noradrenergic control of pupillary activity by the LC is elaborated further in the present review. An anatomical link has been described from the LC to the EWN (127, 128). Furthermore, it has been shown recently that this link operates via stimulation of α_2_-adrenoceptors in the EWN. Liu et al. (60) found that pupil dilation evoked by the electrical microstimulation of the LC, following removal of the SCG, was abolished dose-dependently by the α_2_-adrenoceptor antagonist yohimbine, applied to the EWN. This finding is also consistent with the existence of a direct link between the LC and the EWN, and thus has bearing on a controversy regarding the connection between the LC and the EWN (129). Nieuwenhuis et al. (130) argued that there was no direct connection between the LC and the EWN, and suggested a number of possible “alternative anatomical routes.” However, direct and indirect connections are not mutually exclusive: such parallel connections between autonomic nuclei are known to exist in the autonomic nervous system (131). An example is the projection from the retina to the DRN: both a direct and an indirect connection have been described (see Retinal Inputs to the Dorsal Raphe Nucleus, and Figure 6).

Apart from sending an inhibitory output to the EWN, the LC also inhibits other parasympathetic preganglionic brainstem nuclei (salivatory nuclei, vagal nuclei) (44).

##### Inputs

*Latero-posterior hypothalamus*. Orexinergic neurones in the LHA/PFA project to the LC where they innervate both sympathetic and parasympathetic premotor neurones (see Connections of Excitatory Sympathetic Premotor Neurones). Electrical stimulation of the lateral hypothalamus in experimental animals leads to pupil dilation due to inhibition of the EWN via an inhibitory noradrenergic input (78). The likely mechanism underlying this observation is the activation of parasympathetic premotor neurones in the LC via an excitatory orexinergic input from the hypothalamus, leading to inhibition of the EWN via the stimulation of α_2_-adrenoceptors.

*Amygdala*. The central nucleus of the amygdala sends an excitatory peptidergic projection to the LC; the neuropeptide involved is corticotrophin-releasing factor (CRF) (132). Both the amygdala (133) and the LC (134) have been implicated in the generation of anxiety. Furthermore, there is likely to be a synergistic interaction between these two nuclei in mediating anxious responses: stress-induced activation of the central amygdala is transmitted to the LC by a CRF-containing output from the amygdala (135, 136).

Anxious states amenable for experimental study can be generated by the paradigm of fear conditioning: pairing of a noxious stimulus (e.g., electric shock) with a neutral stimulus (e.g., light, sound) leads to the development of the ability of the neutral stimulus to evoke the response to the noxious stimulus. Using this paradigm, it was possible to modulate two physiological reflexes, the acoustic startle reflex and the pupillary light reflex, by the anticipatory anxiety associated with the procedure (43). Interestingly, the two reflexes are changed in opposite directions: the acoustic startle reflex is potentiated, whereas the pupillary light reflex is inhibited by conditioned fear.

In the case of the modulation of both reflexes by fear (“anticipatory anxiety”), the amygdala and the LC play a joint role. The activation of the amygdala by stressful stimulation is transmitted to the LC, leading to potentiation of the noradrenergic facilitation of striated muscle contraction, in the startle reflex paradigm, and enhancement of the noradrenergic inhibition of the EWN, leading to inhibition of the light reflex (43). As the inhibition of the light reflex by fear cannot be antagonized by the topical application of the α_1_-adrenoceptor antagonist dapiprazole, while a small associated increase in pupil diameter can (137), the fear-inhibited light reflex is likely to be mediated entirely by the parasympathetic output to the iris, consistent with the predominant activation of parasympathetic premotor neurones in the LC by fear. The sensitivity of the accompanying mydriasis to antagonism by dapiprazole would indicate the associated involvement of the sympathetic output, probably arising from the activation of sympathetic premotor neurones in the LC.

*Collaterals from spinothalamic tract*. Collaterals from the spinothalamic tract project to parasympathetic premotor neurones in the LC in nocturnal animals, such as rats (40, 125, 126), and in crepuscular animals, such as cats (40, 78). Via this pathway, pain signals cause excitation of parasympathetic premotor neurones projecting to the EWN, where stimulation of inhibitory postsynaptic α_2_-adenoceptors would lead to parasympathetic inhibition and pupil dilation (Figure 5).

#### Association With Sleep/Arousal Network

The sleep/arousal network is an assembly of interacting nuclei in the hypothalamus and brainstem, responsible for the control of arousal (6). The network consists of functionally antagonistic wake-promoting nuclei (WPN) and sleep-promoting nuclei (SPN). WPN are active during wakefulness and quiescent during sleep, whereas SPN display the opposite pattern of activity. Each nucleus in the network is defined by its connections and the neurotransmitter used. WPN neurones use glutamate, the neuropeptide orexin, acetylcholine, and the monoamines noradrenaline, dopamine, serotonin (5-hydroxytryptamine [5-HT]), and histamine. The neurotransmitters of SPN neurones are γ-amino-butyric acid (GABA) and galanin. WPN send excitatory outputs to the cerebral cortex, whereas SPN function mainly by inhibiting WPN.

The LC is in the hub of this network. It receives inputs from all nuclei of the network, both WPN and SPN, and thus is a major integrator of sleep/arousal-related neuronal activity. It sends excitatory outputs, that operate via α_1_-adrenoceptors, to the cerebral cortex and other wake-promoting nuclei, and inhibitory outputs, that operate via α_2_-adrenoceptors, to the SPN (Figure 2). The LC has a two-way, mutually antagonistic relationship with the VLPO of the hypothalamus, a major sleep-promoting nucleus. The LC inhibits the VLPO, thus releasing other wake-promoting nuclei (e.g., tuberomamillary nucleus, TMN) from GABAergic inhibition, and consequently promoting waking, whereas the VLPO inhibits the LC, and thus facilitates sleep (Figure 4) (42).

Arousal-modifying drugs (sedatives, anesthetics, stimulants) may influence LC activity either directly or indirectly via inputs from the sleep/arousal network. Directly acting sedative drugs are the α_2_-adrenoceptor agonists (e.g., clonidine, dexmedetomidine) and the μ opiod receptor agonists (e.g., morphine, heroin). The α_2_-adrenoceptor agonists “switch off” LC activity by stimulating inhibitory autoreceptors on LC neurones (see α2-Adrenoceptors Associated With Premotor Autonomic Neurones). The μ opiod receptor agonists also suppress LC activity since μ opiod receptors are co-localized with α_2_-adrenoceptors in the membrane of LC neurones and operate via a shared signaling mechanism (i.e., blockade of potassium channels) (138). Examples of drugs indirectly influencing LC activity and thus leading to alterations in the level of arousal are the stimulant modafinil and the sedative drug pramipexole. Both drugs act via an excitatory dopaminergic pathway from the VTA to the LC (“mesocoerulear pathway”). Modafinil increases dopaminergic excitation of LC neurones via blocking the uptake of dopamine into dopaminergic nerve terminals, leading to stimulation of excitatory postsynaptic D_2_ dopamine receptors (67), whereas pramipexole reduces LC activity by attenuating the dopaminergic activation of LC neurones via stimulating inhibitory D_2_ dopamine autoreceptors on the dopaminergic neurones (139).

As the LC is not only a wake-promoting, but also a pre-autonomic nucleus, it couples arousal and autonomic activity (“arousal/autonomic activity interphase”) (42, 68). Consequently, alterations in the level of arousal are transmitted to the pupil. In general, an increase in alertness is associated with an increase in sympathetic output and pupil dilation, whereas sedation is linked to a decrease in sympathetic output and pupil constriction.

During transition from wakefulness to sleep (drowsiness or sleepiness), there is likely to be instability between opposing excitatory wake-promoting and inhibitory sleep-promoting inputs impinging on the LC, leading to fluctuations in LC activity. Due to the close association between LC activity and pupillary diameter (see Noradrenergic Pathway), the drowsiness-related fluctuations of LC activity are transmitted to the pupil, leading to fluctuations in pupil diameter. Thus instability between opposing excitatory and inhibitory inputs may be the basis for the appearance of pupillary oscillations in darkness, termed “fatigue waves” or “sleepiness waves” (140, 141). These oscillations increase as the level of arousal decreases. The Pupillographic Sleepiness Test (PST) provides quantitative measures of the oscillations that can be used as indices of the degree of sedation (142, 143). Indeed, the two measures of pupil diameter fluctuations generated by the PST (total power, Pupillary Unrest Index), correlate with electroencephalographic and subjective measures of sedation (144, 145).

The PST has also been used to assess the sedative and alerting properties of centrally acting drugs (74, 119, 139, 146–148), and the PST measures correlate well with other measures of alertness, such as critical flicker fusion frequency (CFFF) and visual analog scales. It would be expected that drug-induced changes in arousal would affect both pupillary indices of alertness: in the case of sedation, an increase in sleepiness waves would be paralleled by miosis, and in the case of stimulation, a decrease in sleepiness waves would be paralleled by mydriasis. Although this prediction has been confirmed in the case of some drugs (e.g., clonidine, yohimbine, modafinil), there are also exceptions to this general pattern. Examples of a dissociation between the effects of sedation on pupillary oscillations and pupil diameter are two highly sedative drugs: diazepam and pramipexole. While both drugs enhance pupillary oscillations in darkness, and display sedative effects on CFFF and visual analog scales, diazepam-induced sedation is associated with no change in pupil diameter (119), whereas pramipexole-induced sedation is accompanied by mydriasis (139). The possible explanation for the dissociation may lie in some actions of these drugs outside the sleep/arousal network or the LC. Such extraneous actions may either compromise the transmission of sympatho-excitatory signals from the LC to the SCG, or change the balance between sympathetic and parasympathetic outputs to the iris, by interfering with the parasympathetic output.

Diazepam, like all benzodiazepines, enhances the effect of endogenously released GABA at inhibitory GABA_A_ receptors (149). Although the LC is richly endowed with GABA receptors, these receptors are insensitive to benzodiazepines (150). Therefore, benzodiazepines would induce sedation by stimulating inhibitory GABA_A_ receptors elsewhere in the sleep/arousal network, leading to a reduction in the activity of wake-promoting neurones. However, the resultant decrease in LC activity is not passed on to the pupil by the sympathetic output from the LC. The possibility that diazepam may alter the relationship between the sympathetic and parasympathetic outputs to the iris has been excluded: it does not affect the parameters of the light reflex response or the diameter of the pupil dilated by the cholinoceptor antagonist tropicamide, applied topically (119). Therefore it is likely that diazepam interferes with sympathetic outflow “downstream” from the LC, most probably at the level of the IML.

Preganglionic sympathetic neurones in the IML integrate inputs from supraspinal premotor neurones, including those in the LC, and an intricate network of intraspinal interneurones (151). GABA receptors have been identified on bulbospinal neurones, projecting to sympathetic preganglionic neurones in the IML, on presynaptic terminals of such neurones, and on interneurones in the IML (152). Indeed, GABAergic neurotransmission plays an important role in controlling sympathetic outflow (153). Benzodiazepines may increase the activity of sympathetic preganglionic neurones via disinhibiting some of their excitatory inputs, and this increase in activity may mask the effect of the sedation-induced reduction in the excitatory input from the LC (119). However, further experimental work would be needed to confirm this hypothesis.

Pramipexole is a dopamine D_2_/D_3_ receptor agonist with a high affinity for inhibitory D_2_ autoreceptors on dopaminergic neurones. It is highly sedative due to inhibiting an excitatory input to the LC from dopaminergic neurones located in the VTA. As expected, pramipexole-induced sedation is associated with enhancement of pupillary sleepiness waves. However, paradoxically, this is paralleled by mydriasis, rather than miosis (139, 148). This paradoxical effect of pramipexole may be due to the unexpected finding that it attenuates the pupillary light reflex response. As pramipexole has no affinity for cholinoceptors, a central mechanism has been postulated. Pramipexole, by stimulating inhibitory D_2_ receptors on dopaminergic neurones in a putative excitatory pathway projecting to the EWN (“meso-pupillomor pathway”), may withdraw the dopaminergic activation of EWN neurones. Consistent with this model, it has been shown that amisulpiride, a D_2_ dopamine autoreceptor antagonist, evokes an effect opposite to that of pramipexole: it potentiates the light reflex (148). Therefore, in the case of pramipexole-induced sedation, miosis resulting from a reduction in sympathetic outflow to the iris, as a consequence of reduced LC activity, may have been superseded by mydriasis, due to parasympathetic inhibition.

In conclusion, a change in pupil diameter may be a reliable index of drug-induced sedation only in the case of drugs that reduce sympathetic outflow to the iris by selectively reducing LC activity, such as the α_2_-adrenoceptor agonists (e.g., clonidine, dexmedetomidine) (63) However, as many sedative drugs also influence sympathetic output by actions outside the LC, and/or also affect parasympathetic output to the iris, sedation-induced pupil diameter changes should not be used to draw conclusions about the sedative properties of centrally acting drugs. On the other hand, as alterations in pupillary oscillations (“sleepiness waves”) are likely to be linked directly to LC activity, they may provide a reliable measure of sedation.

#### Connections Between Sympathetic and Parasympathetic Premotor Neurones

As discussed above (see Functional Organization of Noradrenergic Premotor Autonomic Neurones in the Locus Coeruleus), there is evidence supporting the view that sympathetic and parasympathetic premotor neurones in the LC form separate populations, and, in many situations, operate independently. However, the sympathetic and parasympathetic divisions of the autonomic nervous system do not function in isolation. Examples of cross-talk between the two divisions have been described in the medulla oblongata and the PVN in the hypothalamus (154).

There is also evidence of cross-talk between the two populations of premotor autonomic neurones in the LC. The anatomical basis of such cross-talk may be the gap junctions between LC neurones, through which cells can communicate with each other via eletrotonic transmission (155). Electrotonic coupling of LC neurones has been implicated in the synchronization of spontaneous firing and the generation of endogenous rhythmic activity (156, 157). An alternative mechanism may be the activation of noradrenergic parasympathetic premotor neurones from recurrent excitatory axon terminals of sympathetic premotor neurones. Indeed, such a mechanism has been described to operate in the LC (158).

The following two subsections discuss how two variables, light (Dual Modulation of Autonomic Activity by Light) and noradrenergic drugs (monoamine depletors, reuptake inhibitors, α_2_-adrenoceptor agonists) (Pupillary Effects of Noradrenergic Drugs), may influence autonomic function by interacting with both contingents of noradrenergic premotor neurones in the LC.

##### Dual Modulation of Autonomic Activity by Light

Light is a powerful activator of sympathetic activity, consistent with light-evoked stimulation of sympathetic premotor neurones in the LC (see Dorsomedial hypothalamus). However, in addition to its sympatho-excitatory effect, light has also been reported to evoke a parasympatholytic effect (55, 159). The possible mechanism for this dual effect of light on autonomic outflow, affecting both divisions of the autonomic nervous system, may be the simultaneous activation of both sympathetic and parasympathetic premotor neurones in the LC. Parasympathetic premotor neurones may have been activated either directly or indirectly, via the spread of sympathetic premotor neuronal activity to parasympathetic premotor neurones via electorotonic transmission or recurrent axon collateras.

##### Pupillary Effects of Noradrenergic Drugs

Drugs acting at noradrenergic neurones may modify the activity and/or the transmitted effects of both sympathetic and parasympathetic premotor neurones, leading to alterations in both sympathetic and parasympathetic pupil control. Three classes of such drugs will be considered: vesicular monoamine transporter (VMAT) inhibitors (“monoamine depletors”), noradrenaline reuptake inhibitors, and α_2_-adrenoceptor agonists.

Monoamines are accumulated in synaptic vesicles of the nerve terminals by an active membrane pump, the vesicular monoamine transporter (VMAT). The form of VMAT accumulating noradrenaline is termed VMAT2. Drugs that inhibit VMAT2 in noradrenergic nerve terminals, such as reserpine and tetrabenazine, lead to depletion of noradrenergic neurones of noradrenaline (160). These drugs are not selective for either sympathetic or parasympathetic premotor neurones in the LC: they deplete both populations of noradrenergic premotor neurones of noradrenaline. Depletion of noradrenaline of sympathetic premotor neurones leads to a sympatholytic effect (sedation, miosis, hypotension), whereas depletion of noradrenaline of parasympathetic premotor neurones results in a parasympathomimetic effect (potentiation of the light reflex response, increase in salivation) (161). The parasympathomimetic effect is likely to be due to removal of the noradrenergic inhibition of the EWN by the LC.

Noradrenaline reuptake inhibitors, including a number of antidepressants, enhance the effect of released noradrenaline, by blocking its reuptake into noradrenergic nerve terminals (44). They, like the VMAT inhibitors, are not selective for either population of pre-autonomic noradrenergic neurones in the LC, and potentiate the effects of noradrenaline at all the different targets of noradrenergic projection. The antidepressants desipramine, reboxetine, and venlafaxine enhance both the sympatho-excitatory and parasympatholytic effects mediated by noradrenergic autonomic outputs. These drugs cause mydriasis and shortening of the recovery time of the light reflex response, due to potentiation of noradrenergic stimulation of preganglionic sympathetic neurones in the IML and of the dilator pupillae muscle in the iris. They also attenuate the pupillary light reflex response and reduce salivary output, due to potentiation of the noradrenergic inhibition of preganglionic parasympathetic neurones in the EWN and the salivatory nuclei (162–165). It should be noted that while the parasympatholytic effect of desipramine could be due to the blockade of cholinoceptors in the iris, this explanation cannot be applied to the parasympatholytic effects of venlafaxine and reboxetine since these drugs have little affinity for cholinoceptors (164).

In contrast to the VMAT inhibitors and noradrenaline reuptake inhibitors, α_2_-adrenoceptor agonists, like clonidine and dexmedetomidine, are selective for sympathetic premotor neurones in man and other diurnal species (see α2-Adrenoceptors Associated With Premotor Autonomic Neurones). The basis for this selectivity is likely to be a difference in the baseline activities of sympathetic and parasympathetic premotor neurones. Sympathetic premotor neurones are likely to have high baseline activity due to their stimulation by light via an input from the retina (see Dorsomedial hypothalamus), whereas the baseline activity of parasympathetic premotor neurones is likely to be low. Therefore, while inhibitory α_2_ adrenergic autoreceptors are likely to occur on both populations of premotor autonomic neurones, the low baseline of parasympathetic premotor neurones does not allow the conversion of their stimulation into an inhibitory response (65). Indeed, while α_2_-adrenoceptor agonists consistently evoke miosis, reflecting sympatho-inhibition in man, they do not usually affect the parasympathetically mediated light reflex response. However, there are exceptions to this general pattern: α_2_-adrenoceptor agonists may occasionally potentiate the light reflex response (120, 166), consistent with the attenuation of the inhibition of the EWN by the LC. It is likely that in these cases the baseline activity of parasympathetic premotor neurones was high enough to allow autoreceptor stimulation to be converted into an observable response. The baseline activity of parasympathetic premotor neurones may have been raised by the spread of activity from the sympathetic premotor neurones to the parasympathetic premotor neurones via electronic transmission through gap junctions. An alternative mechanism may be stimulation of postsynaptic α_2_-adrenoceptors on postsynaptic neurones in the EWN by clonidine, leading to disinhibition of the light reflex, as seen in nocturnal animals (see α2-Adrenoceptors Associated With Premotor Autonomic Neurones).

### Serotonergic Pathway

This pathway is displayed in Figure 6. The figure shows the dual sympathetic/parasympathetic innervation of the iris, including the light reflex pathway via the parasympathetic output. The hub of the serotonergic pathway is the dorsal raphe nucleus (DRN) which contains serotonergic neurones, some of which function as sympathetic premotor neurones. These neurones send an excitatory projection to preganglionic neurones in the IML where it stimulates 5HT_2A_ receptors. The premotor autonomic neurones in the DRN contain inhibitory 5HT_1A_ receptors: the stimulation of these receptors by serotonin, released from recurrent serotonergic axon terminals, inhibits the activity of the serotonergic neurones. The existence of parasympathetic premotor neurones has been postulated: these neurones, via an inhibitory output to the EWN, would inhibit the light reflex. However, although the DRN inhibits the parasympathetic output to the pupil, this is likely to be via an indirect route (see 5-HT1A Receptors, below). The DRN also sends an excitatory output to the cerebral cortex where it stimulates 5HT_2A_ receptors. The DRN receives afferents from the retina, both directly and indirectly, via the LHA/PFA.

#### Serotonergic Neurones

Serotonin (5-hydroxytryptamine, 5-HT) is one of the major monamine neurotransmitters that, like noradrenaline, is involved in the regulation of both arousal (6, 167) and autonomic function (168, 169). Serotonergic neurones are located in nine nuclei (B1–B9) in the midline raphe of the brainstem, and project widely throughout the neuraxis (170, 171). Largest of these nuclei is B7, corresponding to the DRN, that is responsible for the serotonergic control of arousal (172). Several serotonergic nuclei are involved in autonomic regulation, including the DRN and a number of caudal raphe nuclei. These nuclei project to the IML of the spinal cord (171), where they are likely to stimulate excitatory 5HT_2_ receptors on sympathetic preganglionic neurones (152). There is also a population of serotonergic interneurones in the IML (173).

Serotonin interacts with a large array of presynaptic (auto) and postsynaptic receptors that can mediate both excitatory and inhibitory effects. 5-HT_1_ receptors are inhibitory, and occur both in presynaptic and postsynaptic locations, whereas 5HT_2_ and 5-HT_3_ receptors are excitatory, and occur postsynaptically (174). The most common and best studied receptor sub-types are the 5-HT_1A_ and the 5-HT_2A_ receptor (175). Inhibitory 5-HT_1A_ autoreceptors on serotonergic neurones play an important role in the regulation of serotonergic neurotransmission (176).

#### Retinal Inputs to the Dorsal Raphe Nucleus

##### Direct Link

During the past decade or so, an anatomical link has been identified between the retina and the DRN (“retino-raphe projection”) in a number of rodent species. These species include the rat (177–179), the mouse (180); the Mongolian gerbil (179, 181, 182), and the Chilean degus (183). It has been shown that stimulation of this pathway by light can modulate the expression of cFos, an index of neuronal activity, in the DRN (182). Furthermore, stimulation of the DRN by light can lead to alterations in complex behaviors, such as affective (184) and defensive (180) behaviors. It has been shown that both conventional and melanopsin-containing retinal ganglion cells project to the DRN (185). The majority of retinal ganglion cells projecting to the DRN are conventional alpha-like ganglion cells with Y-like physiological properties (186, 187).

##### Indirect Link

Apart from the direct link described above, an indirect link via the orexinergic neurones of the LHA/PFA has also been reported. Orexinergic neurones may be directly light-sensitive via an input from the retina (188), or may be activated indirectly by light via the SCN (189). It has been found that, in the diurnal rodent Nile grass rat, a light pulse evoked an increase in the expression of cFos, in both the LHA/PFA and the DRN. Pretreatment of the animals with the orexin receptor type 1 (OXR1) antagonist SB-334867 prevented the activation of the DRN by light, leading to the conclusion that “in the diurnal brain light induces excitatory responses in the 5-HTergic DRN through activating orexinergic pathways” (104). For the role of the orexinergic system in pupillary control, see Latero-Posterior Hypothalamus.

#### 5-HT Receptors Modulating Pupil Function

Serotonergic neurones in the DRN operate via stimulating serotonin receptors both in the DRN and the targets innervated by it. The two most important receptor types are the 5-HT_1A_ and 5-HT_2_ receptor. The role of these receptors in controlling pupil function has been explored using selective 5-HT_1A_ receptor agonists and 5-HT_2_ receptor antagonists.

##### 5-HT_1A_ Receptors

5-HT_1A_ receptors occur both presynaptically (autoreceptors) and postsynatically where they mediate an inhibitory action. The autoreceptors are usually more sensitive than the postsynaptic receptors, and their role in controlling serotonergic neuronal function is analogous to that of the α_2_-adrenoeptors in controlling noradrenergic neurone function (see α2-Adrenoceptors Associated With Premotor Autonomic Neurones). 5-HT_1A_ autoreceptors are abundant on serotonergic neurones in the DRN (190). The stimulation of these receptors on sympathetic premotor neurones would mediate a sympatholytic effect by switching off the activity of these neurones, and thus attenuating their excitatory influence on sympathetic preganglionic neurones (152). Postsynaptic 5-HT_1A_ receptors also play a role in autonomic regulation: by inhibiting sympathetic premotor neurones in the RVLM they mediate a sympatholytic effect on cardiovascular function (168). It has been postulated that, like α_2_-adrenoceptors, 5-HT_1A_ receptors may occur on parasympathetic preganglionic neurones in the EWN (191) (Figure 6).

5-HT_1A_ receptor agonists have marked effects on pupil function that are, like the effects of the α_2_-adrenoceptor agonists, species-specific (see α2-Adrenoceptors Associated With Premotor Autonomic Neurones). In diurnal species, these drugs evoke miosis, whereas in nocturnal animals they cause mydriasis.

The 5-HT_1A_ receptor agonists buspirone, lesopitron, and 8-OH-DPAT evoked dose-dependent miotic responses in rabbit (192), monkey (193), and man (194–196). As in man, the buspirone-induced miosis was unaffected by the topical application of the cholinoceptor antagonist homatropine, it was concluded that the miotic response was likely to be due to sympathetic inhibition (194). Miotic responses to buspirone and lesopitron were also light-dependent: responses were larger in light than in darkness. As miotic responses to the α_2_-adrenoceptor agonist clonidine show the same light-dependence, a similar mechanism was postulated, probably involving the noradrenergic inhibition of the EWN (see Pharmacological Unmasking of Light-Evoked Latent Pupil Dilation) (195). The light-dependence of the miotic responses to the 5-HT_1A_ receptor agonists is consistent with the operation of a serotonergic light-stimulated sympathetic pathway.

The 5-HT_1A_ receptor agonist 8-OH-DPAT evoked consistent dose-dependent mydriatic responses in mice (197) and rats (191). The pupillary responses could be antagonized by not only 5-HT_1A_ receptor antagonists (e.g., WAY-100135 and WAY 100635), but also by α_2_-adrenoceptor antagonists (e.g., yohimbine and RS 79948). These observations argue against the existence of a direct serotonergic inhibitory input to the EWN operating via 5-HT_1A_ receptors (Figure 6). It was proposed that 8-OH-DPAT might have acted indirectly via the noradrenergic system: activation of noradrenergic neurones in the LC by the drug would have increased the release of noradrenaline onto inhibitory α_2_-adrenoceptors in the EWN (191). Indeed, an intricate neuronal network has been proposed to operate within the LC modulating the firing of noradrenergic neurones. In this network, the noradrenergic neurones may be under tonic inhibition by GABAergic interneurones that in turn may be inhibited by a serotonergic input operating via inhibitory 5-HT_1A_ receptors. Therefore, disinhibition of the noradrenergic neurones by 5HT_1A_ receptor stimulation could lead to an increase in noradrenergic neuronal firing (198).

##### 5-HT_2_ receptors

An ascending output from the DRN to the cerebral cortex stimulates excitatory 5-HT_2A_ receptors, and thereby increases arousal (6), and a descending output to the sympathetic preganglionic neurones in the IML stimulates 5-HT_2A_ receptors, leading to sympathetic stimulation (Figure 6). It has been shown that the 5-HT_2_ receptor antagonists ICI 169,369 and ICI 170,809 have dose-dependent miotic and sedative effects in man (199, 200), consistent with the attenuation of 5-HT_2_ receptor-mediated functions. The dose-dependent miosis suggests that the 5-HT_2_ receptors in the IML may mediate a tonic sympatho-excitatory effect on the pupil.

## Conclusions

Light has robust effects on the autonomic control of the pupil: it stimulates the parasympathetic output and inhibits the sympathetic output. Stimulation of the parasympathetic output results in the light reflex mediating a constrictor response, whereas sympathetic inhibition, working “in the background,” allows unimpeded expression of light-evoked pupil constriction (1). While the mechanisms underlying the light reflex have been the subject of intensive investigation, especially since the discovery of the role of melanopsin-containing retinal ganglion cells in its initiation (201), there has been relatively less interest in the sympathetic control of the pupil by light.

Interestingly, there may be multiple sympathetic pathways mediating the effect of light on the pupil: two pathways mediating an inhibitory effect (“light-inhibited sympathetic pathways”) and two pathways mediating a paradoxical stimulatory effect (“light-stimulated sympathetic pathways”) are described.

While the inhibitory effect of light on the sympathetic output to the pupil was demonstrated in the 1960s and 1970s, little experimental work has been done since then. Okada et al. (13) demonstrated a connection between the pretectal area and the sympathetic preganglionic neurones projecting to the iris. The course of this pathway has not been investigated since then. However, review of the literature of experimental work investigating the connections of the pretectum to autonomic nuclei, allows filling in the missing gaps. This has led to the proposal of the pretectum/periaqueductal gray pathway (see Pretectum/Periaqueductal Gray Pathway). A second putative light-inhibited sympathetic pathway is the suprachiasmatic nucleus/paraventricular nucleus pathway (see Suprachiasmatic Nucleus/Paraventricular Nucleus Pathway). This pathway overlaps with the pathway controlling melatonin synthesis, as sympathetic preganglionic neurones in the same segments (C8-T2) of the IML innervate, via the SCG, both the pineal gland and the dilator pupillae muscle in the iris. Although the role of this pathway in mediating the effect of light on the sympathetic control of melatonin synthesis is well established, its role in mediating the inhibitory effect of light on the sympathetic output to the pupil has not been studied experimentally.

The two light-stimulaed sympathetic pathways are based on well-established connections of their “hub” nuclei, the LC and the DRN. Both these nuclei are light sensitive, either directly (DRN) and/or indirectly (DRN and LC), and there is evidence of their roles in both the sympathetic and parasympathetic controls of the pupil. Light has a manifest sympatho-excitaroy effect on functions (e.g., cardiovascular or renal activity) controlled by the thoraco-lumbar sympathetic outflow. However, at the levels of C8-T2, the sympatho-excitatory effect of light may be superseded by its powerful inhibitory effect required for the operation of the light reflex and control of melatonin synthesis. Therefore, pupil dilation resulting from the stimulation of the sympathetic output to the iris would be masked by the pupil-constricting effect of light. The latent mydriasis can be unmasked by drugs that modulate the activity of the hub nuclei. Drugs that inhibit LC activity (e.g., clonidine, diphenhydramine) or DRN activity (e.g., buspirone) potentiate light-evoked pupil constriction, while drugs that enhance LC activity (e.g., yohimbine, modafinil) antagonize light-evoked pupil constriction. The light-stimulated sympathetic pathways, by attenuating light-evoked pupil constriction, may enable diurnal animals to function in daylight, when light may cause pinpoint pupils in nocturnal animals (202).

The activity of the light-stimulated pathways appears to be related to age. The monotonic decline in pupil diameter with increasing age in humans (203–205) may reflect the gradual withdrawal of the activity of the light-stimulated sympathetic pathways since the decline in pupil diameter is paralleled by the age-dependent decline in the number of noradrenergic neurones in the LC (42). The effect of age on the pupil is accentuated in Alzheimer's disease (206) when the loss of noradrenergic neurones in the LC exceeds that seen in old age (207).

The noradrenergic light-stimulated sympathetic pathway has widespread connections via sympathetic and parasympathetic premotor neurones in the LC, and via these connections it is integrated into the wider central autonomic network (41, 44). It is also integrated with the sleep/arousal network, and participates in the processing of pain signals and fear/anxiety. Many drugs (sedatives, stimulants, antidepressants, anxiolytics) modify pupil function by actions via the noradrenergic light-stimulated sympathetic pathway. Through its multiple inputs the noradrenergic light-stimulated sympathetic pathway is amenable to modulation by a wide range of physiological and psychological variables, and via its outputs it can transmit sympathetically and parasympathetically mediated alterations in pupil function.

There is a remarkable species difference in the operation of light-stimulated sympathetic pathways: diurnal animals respond differently from nocturnal animals to light, noxious stimulation, and autoreceptor agonist drugs (e.g., clonidine in the noradrenergic, buspirone in the serotonergic light-stimulated sympathetic pathway). A tentative explanation for the species difference may be that it is related to regular exposure to light in diurnal animals that may lead to proliferation and/or a raised baseline activity of sympathetic premotor neurones in the LC and DRN. Therefore autoreceptor agonists and pain signals may affect sympathetic premotor neurones preferentially, as compared to parasympathetic premotor neurones, in diurnal animals. These observations suggest that the effects of some non-luminance-related variables (monoaminergic autoreceptor agonists, noxious stimuli) may be influenced by the luminance-exposure history of the species, determined by the “temporal niche.”

Apart from transmitting slow time-course (“tonic”) changes in pupil diameter in response to light, the LC is also involved in mediating non-luminance-related fast time-course (“phasic”) pupil dilations in response to cognitive load (57). It has been shown in the monkey (58, 109) and in the mouse and rat (59) that cognitive load, applied using different paradigms, evokes fast transient changes in the firing rate and pattern of LC neurones. Furthermore, corresponding changes can be observed in neuronal firing in different areas of the cerebral cortex and colliculi.

Unraveling the multiple sympathetic pathways controlling the pupil suggests that the sympathetic has roles beyond fading away in the background when the light reflex operates. While the parasympathetic pathway mediating the light reflex has one robust dedicated function, the sympathetic pathways, through their connections, are multifunctional, integrating pupil function with a wide range of autonomic, neuroendocrine, physiological, and psychological functions.

## Author Contributions

The author confirms being the sole contributor of this work and has approved it for publication.

### Conflict of Interest Statement

The author declares that the research was conducted in the absence of any commercial or financial relationships that could be construed as a potential conflict of interest.

## Abbreviations

CFFF: critical flicker fusion frequency
CRF: corticotropin-releasing factor
DMH: dorsomedial hypothalamus
DRN: dorsal raphe nucleus
IML: intermedio-lateral column (of spinal cord)
EWN: Edinger-Westphal nucleus
GABA: y-aminobutyric acid
GC: ganglion ciliare
5-HT: 5-hydrytryptamine (serotonin)
LC: locus coeruleus
LHA: lateral hypothalamic area
OPN: olivary pretectal nucleus
PAG: periaqueductal gray (matter)
PFA: perifornical area
PVN: paraventricular nucleus
SCG: superior cervical ganglion
SCN: suprachiasmatic nucleus
SPN: sleep-promoting neurones
TMN: tuberomamillary nucleus
VLPO: ventrolateral preoptic nucleus
VMAT: vesicular monoamine transporter
VTA: ventral tegmental area
WPN: wake-promoting neurones.
